# No Evidence of Trade-Off between Farm Efficiency and Resilience: Dependence of Resource-Use Efficiency on Land-Use Diversity

**DOI:** 10.1371/journal.pone.0162736

**Published:** 2016-09-23

**Authors:** Helena Kahiluoto, Janne Kaseva

**Affiliations:** 1 Lappeenranta University of Technology, Saimaankatu 11, 15140 Lahti, Finland; 2 Natural Resources Institute Finland, 31600 Jokioinen, Finland; Shanxi University, CHINA

## Abstract

Efficiency in the use of resources stream-lined for expected conditions could lead to reduced system diversity and consequently endanger resilience. We tested the hypothesis of a trade-off between farm resource-use efficiency and land-use diversity. We applied stochastic frontier production models to assess the dependence of resource-use-efficiency on land-use diversity as illustrated by the Shannon-Weaver index. Total revenue in relation to use of capital, land and labour on the farms in Southern Finland with a size exceeding 30 ha was studied. The data were extracted from the Finnish Profitability Bookkeeping data. Our results indicate that there is either no trade-off or a negligible trade-off of no economic importance. The small dependence of resource-use efficiency on land-use diversity can be positive as well as negative. We conclude that diversification as a strategy to enhance farm resilience does not necessarily constrain resource-use efficiency.

## Introduction

Evidence-based policy may be wishful thinking in times of turbulence and multi-dimensional epistemic and ontological uncertainty. Robust strategies [1], which work well even if the information is imperfect or inputs to the system vary [2], may yield a more favorable cost-benefit ratio for societal investments [3]. Enhancement of system resilience is one such robust strategy [2], and therefore currently an important complementation to add to efficiency, for sustainability of farming.

Resilience is the capacity of a system to tolerate disturbance and reorganize while retaining its function, structure and identity [4–6], and to shape change and learn [7–8]. If a social-ecological system threatens resilience at larger scales, transformational change is required [9]. In the face of increased turbulence in the global climate and markets, the resilience discourse has emerged in international environmental and economic policy since the start of the current decade identity, e.g., [10–12]. Increasing effort has also been addressed to mathematically model various aspects of stability of complex systems, e.g., [13–16]. Diversity generates a variety of possible responses to variability [17–19] and to various threats [20] and material for transformation [21], and as such is a prerequisite for system resilience [20, 22–26]. Diversity also implies the generation of ‘perpetual novelty’ [26], which is critical for reorganizing the system after disturbance [27].

Efficiency is a key economic concept and has made a crucial contribution to sustainability discourses across disciplines and sectors [28]. Eco-efficiency, in terms of the efficient use of resources (‘more from less’), has long dominated the interpretation of sustainability [29–30] and has substantially influenced societal development strategies. Increasing resource-use efficiency, implying a small ratio of resources to products or revenue, e.g., higher yields per unit area of land or other natural or economic resources in agricultural systems, is believed to promote economic performance, food security and environmental protection [31–32]. Korhonen and Seager [33], Ulanowicz et al. [34] and Goerner et al. [35] argued for the complementarity of the two perspectives, i.e., efficiency and resilience, in sustainable development.

In stable times, system efficiency is streamlined for expected conditions, often creating systems with less diversity, as exemplified by the development at various levels of agricultural systems in industrial countries in recent decades [35–42]. Diversity increases stability of production in variable agricultural environments [43], and land-use diversity appears to increase farm resilience [44] also in terms of economic returns [45–46]. Consequently, efficiency may run counter to system resilience [33], especially through loss of response diversity [18,20]. On the other hand, based on ecological models with economic relevance, Tilman et al. [47] concluded that diversity should enhance efficiency in the use of limited resources.

The relationship of economic performance and biodiversity has been assessed [45–46], especially from the viewpoint of ecosystem services [48–50]. Further, eco-efficiency in terms of products relative to emissions has been related to diversity in what-if scenarios of social-ecological systems [51]. However, empirical evidence for the dependence between resource-use or economic efficiency and production diversity is scarce or non-existent. This knowledge gap is also practically important, because the current understanding of a trade-off relation between economic efficiency and diversity in farming informs agricultural policies.

Inspired by the model-based study of Tilman et al. [47], we tested in an empirical case the general belief that diversity reduces efficiency, e.g., [44]. The aim of this study thus was to investigate empirically, whether there is trade-off between diversity (critical for system resilience) and efficiency of resource-use (also required for sustainability) on farms, and that way contribute to bridging the knowledge gap. We tested the hypothesis that land-use diversity is negatively related to farm efficiency in terms of revenue per unit of land, labour and capital. We tested this hypothesis in the context of Finnish farms, which during the two last decades increased rapidly in specialization and size to achieve greater resource-use efficiency through economies of scale [52–53], and for which resilience is of paramount importance, due to the northernmost location in the world and therefore a rapid climate change, as well as tight linkages to volatile global food markets.

## Materials and Methods

### Farm data

The empirical data analysed here originate from the Finnish profitability bookkeeping data used to compile the Finnish data for the European Farm Accountancy Data Network (FADN), which is maintained by the European Commission. For comparability and access, variable definitions similar to those in FADN were used for the accounting years of 1998–2008 ([54]; http://ec.europa.eu/agriculture/rica/definitions_en.cfm). FADN is used throughout Europe to evaluate income from agricultural holdings and the impacts of the Common Agricultural Policy (CAP). Finland has four FADN regions, but the climatic conditions for agriculture in the north clearly differ from those for the south, and most agricultural production, accounting farms and land-use diversity, is concentrated in southern Finland. Other areas were thus excluded from the analysis to remove the bias that could arise through independent effects of climate on the revenue and on the diversity of agricultural land-use. In addition, available agricultural area restricts land-use diversity. For smaller farms also other reasons of farming (recreation, maintenance of land value, life style, emotional reasons such as heritage etc.) are in a bigger role which could cause bias into the conclusions if such farms would be included in the analysis of the trade-off between resource-use efficiency and diversity (reflecting the relation between economic efficiency and resilience), because such relation has no relevance for the farmers in those cases. Therefore, the utilised agricultural area (UAA) below 30 hectares (ha) was set as the lower limit for farm size in our analysis. After these restrictions were applied to remove obvious sources of bias, we were left with the empirical data for 3 268 farms totally over the years (Table 1).

**Table 1:** Farm inputs (labour, capital and land), total revenue and land-use diversity per production line.

Variable	Mean	St. Dev.	Min	Max
*All farms (3268)*				
Labour, h	3 347	2 144	159	16 608
Farm capital, €	277 055	237 930	19 724	2 288 832
UAA, ha	71	40	30	655
Total revenue, €	90 391	98 649	125	1 222 089
Shannon index	0.686	0.241	0	1.316
*Cereals*, *oilseeds and protein crops (1140)*				
Labour, h	1 686	972	191	8 570
Farm capital, €	180 394	137 295	19 724	2 288 832
UAA, ha	79	52	30	655
Total revenue, €	38 702	34 329	125	419 030
Shannon index	0.675	0.244	0	1.316
*Field crops (453)*				
Labour, h	2 811	1 858	159	12 989
Farm capital, €	227 728	164 551	34 528	983 156
UAA, ha	73	38	31	315
Total revenue, €	75 594	66 440	2 112	454 462
Shannon index	0.869	0.177	0	1.256
*Specialist dairying (692)*				
Labour, h	5 623	2 037	1 982	16 608
Farm capital, €	348 065	301 236	70 732	1788 464
UAA, ha	60	26	30	200
Total revenue, €	123 574	67 223	29 736	393 392
Shannon index	0.648	0.174	0	1.076
*Specialist granivores (284)*				
Labour, h	4 349	1 674	668	14 140
Farm capital, €	482 851	354 022	65 385	2 069 981
UAA, ha	60	27	30	181
Total revenue, €	215 821	161 085	48 569	669 621
Shannon index	0.502	0.252	0	1.054
*Field crops and grazing livestock (283)*				
Labour, h	4 336	1 572	246	10 434
Farm capital, €	255 716	152 461	50 305	924 458
UAA, ha	76	39	30	226
Total revenue, €	64 566	42 936	13 624	173 232
Shannon index	0.781	0.214	0	1.207
*Various crops and livestock (416)*				
Labour, h	3 342	1 208	980	8 091
Farm capital, €	351 556	201 211	74 132	1 161 327
UAA, ha	70	27	30	201
Total revenue, €	127 904	101 417	13 123	927 783
Shannon index	0.641	0.253	0	1.299

UAA = utilised agricultural area; Shannon index = Shannon index for land-use diversity; sample size in parentheses.

### Land-use diversity

The Shannon-Weaver index [henceforth, the Shannon index] [55], the most commonly used diversity index, was used to illustrate farm land-use diversity. Specifically, the Shannon index was used to describe the number and proportional area distribution (richness and evenness) of eight farm land-use types. A Shannon index equal to zero indicates that the farm comprises only one land-use type; the value of the Shannon index increases as the number of different land-use types and/or their evenness increases. The Shannon index gives an equal weight to each observation and is comparable among cases with different compositions [56]. The Shannon index was calculated according to the following eq (1):

$$H=-\sum_{k=1}^{K}\frac{w_{ik}}{W_{i}}\;ln\frac{w_{ik}}{W_{i}},\text{ for }i=1,\ldots ,\;n\text{ farms}$$ (1)

where *k* = *1*, *…*, *K* referring to the number of land-use types; *w*_*ik*_ is the area covered by land-use type *k* of farm *i*; *W*_*i*_ represents the total area of farm *i*; and *w*_*ik*_*/w*_*i*_ is the proportion of area covered by land-use type *k*. The Shannon index is expressed in logarithmic form, and to describe the true diversity (‘land-use diversity’), it needs to be converted (exp(H)).

Agricultural specialisation is a categorical variable in the Finnish profitability bookkeeping data (as in FADN); it consists of categories that are both exhaustive and mutually exclusive such that each observation is assigned to one and no more than one category. The following six agricultural land-use types were used as independent classes for calculating the Shannon index: cereals (corresponding to FADN variable SE035), other field crops (SE041), vegetables, berries, flowers and ornamental plants (SE046*-SE046**), perennial crops (SE054-55), fodder crops and fallow (SE071-73), and other. Only on 69 farms the class ‘other’ represented more than 10% of the agricultural land area, while 89% from the total 3 268 farm observations over years did not have the class ‘other’ at all. This indicates that no bias in the analysis was caused by the lacking information of the diversity within the class ‘other’. We then calculated Pearson’s correlation coefficients for the Shannon index and farm input/output variables, such as UAA, labour, farm capital and total revenue.

### Resource-use efficiency

Resource-use efficiency was measured as a relation between the use of the major farm resources land, labour and capital, and farm revenue, using Cobb-Douglas regression model. In addition, ‘technical efficiency’ was measured with stochastic frontier models as the ratio between the observed output (here total revenue) to the maximum output under the assumption of fixed inputs [57], i.e., the resource-use efficiency of individual farms relative to the maximum resource-use efficiency of the farms. Use of the major farm resources land, labour and capital was illustrated by the following input resources: the total UAA of holding (ha; SE025), total labour input on holding in hours (h; SE011), and farm capital as the sum of the average of the working capital of livestock, permanent crops, land improvements, buildings, machinery and equipment, and circulating capital (€; SE510). The total revenue was calculated as output from crops and crop products, livestock and livestock products and other output (corresponding to FADN variable SE131), in €. For detailed definitions of the variables, see [54].

### The Cobb-Douglas regression model

The Cobb-Douglas production function see [58] is widely used in econometrics to represent the relation between several inputs and production [59]. It allows the quantity of one input to affect the productivity of another input. We included the Shannon index in the model, in analogy to inputs [60]. The Cobb-Douglas model included three inputs (land, capital and labour), the Shannon index, and a single output, total revenue. The model can be expressed as

$$y_{j}=A\;x_{1j}^{\beta_{1}}\;x_{2j}^{\beta_{2}}\;x_{3j}^{\beta_{3}}\;e^{\omega H_{j}+\varepsilon_{j}},\;\;j=1,\ldots ,n$$ (2)

where y_j_ is the output of farm *j* and *x*_*1*_, *x*_*2*_ and *x*_*3*_ are the area (ha), labour (h) and farm capital (€) of farm *j*. The parameter *H*_*j*_ is the Shannon index of farm *j*, and *ε*_*j*_ is the error term, which was assumed to be independent and normally distributed. The remaining parameters (*A*, *β*_*1*_, *β*_*2*_, *β*_*3*_ and *ω*) were unknown and had to be estimated. The equation was modified for estimating the coefficients of the parameters. Taking the natural logarithm of both sides of the equation leads to

$$ln\;y_{j}=ln\;A+\beta_{1}\;ln\;x_{1j}+\beta_{2}\;ln\;x_{2j}+\beta_{3}\;ln\;x_{3j}+\omega H_{j}+\varepsilon_{j},\;\;j=1,\ldots ,n$$ (3)

By choosing *Y = ln(y*_*j*_*)*, *X = ln(x*_*ij*_*)* and *H = H*_*j*_, we obtained a linear regression equation

$$Y=\beta_{0}+\beta_{1}X_{1}+\beta_{2}X_{2}+\beta_{3}X_{3}+\omega H+\varepsilon$$ (4)

where the unknown parameters could be estimated.

Because the production lines differed in terms of diversity and revenue, we included the production lines in the model as dummy variables. Dummy variables are a series of binary variables that identify whether or not each observation is a member of a specific category. The years with a statistically significant (α = 0.1) interaction of Shannon index and production line were not included in further analyses due to technical and interpretational complexities.

### The stochastic frontier production models

The Cobb-Douglas regression models used in the analyses above assume that all farms represent equal technical efficiency. Because this assumption may not be valid, we also applied a stochastic frontier production function, which adds to the model a new term, technical inefficiency that after a mathematical transformation represents the technical efficiency of each farm.

We used two most common stochastic production functions: the Cobb-Douglas and the translog production function. The translog production function is more flexible, because all second order cross-terms of inputs and the Shannon index are included in the model, unlike the Cobb-Douglas production function that assumes all cross-terms to be zero. The stochastic frontier production model included three inputs (land, capital and labour), the Shannon index and a single output, i.e., total revenue. We also investigated whether the association of the total revenue and the Shannon index depends on production line (Table 1) by including a separate intercept term for them.

**Fig 1:**
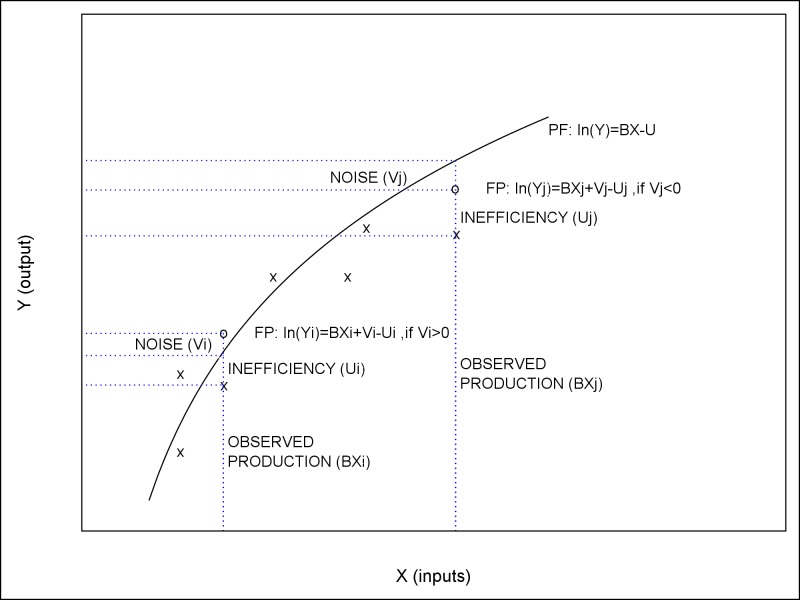
The stochastic production frontier [61–62]. Observed productions and frontier productions are indicated with *x* and *o*, respectively. The frontier production (FP), consisted of observed production, inefficiency effect and random noise, can lie above or below the frontier prodution function (PF), depending on the noise effect.

Thus, the Cobb-Douglas production function can be expressed as

$$ln\;y=\beta_{0}+\sum_{i=1}^{n}\beta_{i}\;ln\;x_{i}+\varepsilon$$ (5)

and the translog production function as

$$ln\;y=\beta_{0}+\sum_{i=1}^{n}\beta_{i}\;ln\;x_{i}+\frac{1}{2}\sum_{i=1}^{n}\sum_{j=1}^{n}\beta_{ij}\;ln\;x_{i}\;ln\;x_{j}+\varepsilon$$ (6)

where *y* is the output of farms and *n* is the number of inputs added with the Shannon index (*x*). The parameters *β*_*0*_, *β*_*i*_ and *β*_*ij*_ are the unknown parameters to be estimated. The *ε = v-u*, where *v* is the systematic error component, which is assumed to be independently and identically distributed, random error having normal distribution with mean being zero and variance being *σ*_*v*_^*2*^. *u* is a non-negative random variable, which is assumed to account for technical inefficiency in production, having normal distribution with mean being zero and variance being σ_u_^2^ (Fig 1).

### Model comparisons

We determined whether the technical inefficiency term needs to be added, relative to the Cobb-Douglas regression models, i.e., whether stochastic frontier models (Cobb-Douglas or translog stochastic frontier models) would be required instead of Cobb-Douglas regression models (see above). The key parameter to test the need of the technical inefficiency term is *γ = σ*_*u*_^*2*^*/(σ*_*u*_^*2*^*+σ*_*v*_^*2*^*)*, which tells the proportion of variance of the efficiency term from the overall variance. If hypothesis H_0_: *γ* = 0 holds, there is no need for an efficiency term in the model. Since *γ ϵ* [0,1] and the hypothesis is one-sided, we used critical values from Kodde and Palm (1986) for a likelihood ratio test. The distributions of a technical inefficiency term were compared based on the information criteria (AIC, Bayesian information criterion (BIC)).

The likelihood ratio test and BIC were used to determine whether the translog production function would be more appropriate than the Cobb-Douglas production function in the stochastic frontier models. The likelihood ratio test statistic can be defined by

$$D=-2\left\{ln\left[L(H_{0})\right]-ln\left[L(H_{1})\right]\right\}$$ (7)

where *L(H*_*0*_*)* and *L(H*_*1*_*)* are the values of the likelihood function of the null hypothesis (*H*_*0*_: *β*_*ij*_ = 0) and the alternative hypothesis. Test statistic *D* is distributed chi-squared with degrees of freedom equal to the number of parameters that are constrained. BIC can be defined by

$$BIC=-2\left\{ln\left[L(H_{i})\right]+k\cdot ln\left[N\right]\right\}\;\;,\;i=0,1$$ (8)

where *k* is the number of free parameters and *N* is the sample size.

To facilitate the interpretation of the translog production models, all the variables were divided by their sample means before estimation. Consequently, the first-order coefficients can be interpreted as elasticities of the sample means [63]. The interaction terms show how the estimated elasticities vary with a movement away from the sample means.

The statistical analyses were performed using SAS software **(**SAS Institute, Inc., Cary, NC, USA**)** and the REG, GLM, MIXED and QLIM procedures. The R 3.1.1 package ‘frontier’ (version 1.1–0) was also used to test more complex models that could not be applied using SAS (R Development Core Team).

## Results

### Land-use diversity in relation to farm inputs and total revenue

The Shannon index varied by farm production line (Table 1); the highest diversity indices were found for farms with field crops as the production line, followed by farms with field crops and grazing livestock. The lowest Shannon index was measured for specialist granivore farms. Only five farms had five different land-use types; the greatest proportion of the farms (55%) had three different land-use types, and forty farms had only a single land-use type. The Shannon index for farm land-use diversity was weakly negatively correlated with total revenue, while it was weakly positively correlated with UAA (Table 2). However, in production-line-specific analyses, the Shannon index was correlated with UAA only, i.e., weakly positively correlated with UAA for cereals, oilseeds and protein crops (r = 0.27, *P*<0.001) and field crops (r = 0.28, *P<*0.001) farms.

**Table 2:** Pearson correlation matrix of land-use diversity, total revenue and inputs (labour, farm capital and land).

	Shannon index	Total revenue	UAA	Labour	Farm capital
Shannon index	1				
Total revenue	-0.102*	1			
UAA	0.201*	0.276*	1		
Labour	-0.029	0.532*	0.162*	1	
Farm capital	-0.104*	0.835*	0.482*	0.561*	1

Shannon index = Shannon index for land-use diversity; UAA = utilised agricultural area; n = 3268. Statistically significant correlations (*P*<0.05) are marked with asterisks.

### Land-use diversity in relation to farm resource-use efficiency

#### Cobb-Douglas regression model

When using the traditional Cobb-Douglas regression models, we found no statistical support for the negative dependence of resource-use efficiency on land-use diversity. There was no statistically significant difference in the dependence of resource-use efficiency on land-use diversity among the production lines; p-values of the Shannon index varied from 0.48 to 0.83 over years. In the fittest model which included the three inputs (land, labour and capital), the Shannon index and the production lines, the estimate of the Shannon index varied from -0.085 to 0.028 with the confidence interval from [-0.323, 0.153] to [-0.206, 0.263], respectively. The models explained 80–86% of the total variance, while the proportion of land-use diversity was less than one percent. In the year 2000, the traditional Cobb-Douglas regression model where the difference among farms in resource-use efficiency is included in the experimental error term, was shown to be adequate, but in other years stochastic frontier production models were preferred (Table 3).

**Table 3:** Comparison of the Cobb-Douglas regression (H_0_) and stochastic frontier Cobb-Douglas (H_1_) and translog (H_2_) production models.

	Log-likelihood values of the models			Comparison of the models		
Year	H_0_: Cobb-Douglas, λ = 0^a^	H_1_: Cobb-Douglas^b^	H_2_: translog^c^	H_0 vs_ H_1_: $\chi_{\;1}^{2}$ ^d^	H_1 vs_ H_2_: $\chi_{\;10}^{2}$ ^e^	H_1 vs_ H_2_: ΔBIC^f^
2000	-123.7	-122.5	-107.1	2.4	30.8*	27*
2001	-147.2	-130.2	-102.1	34.0*	56.2*	1
2002	-128.1	-119.7	-93.6	16.8*	52.2*	4
2004	-159.1	-154.9	-148.6	8.4*	12.6	44*
2005	-172.4	-154.8	-135.5	35.2*	38.6*	18*
2006	-189.1	-140.8	-134.1	96.6*	13.4	43*

Likelihood ratio test (H_0 vs_ H_1;_ H_1 vs_ H_2_) and Bayesian information criterion (H_1 vs_ H_2:_ ΔBIC) were used in statistical inference to select the adequate model for each year. Based on the likelihood ratio test, the Cobb-Douglas regression model is adequate in 2000 and the stochastic frontier Cobb-Douglas production model in 2004 and 2006. Based on the Bayesian information criterion, the stochastic frontier Cobb-Douglas production model is adequate for every year.

^a^ The log-likelihood value of the Cobb-Douglas regression model without the inefficiency effect (λ).

^b^ The log-likelihood value of the stochastic frontier Cobb-Douglas production model with the inefficiency effect.

^c^ The log-likelihood value of the stochastic frontier translog production model with the inefficiency effect.

^d^ Likelihood ratio test for H_0_: The technical inefficiency effect is absent. The significance level α = 0.05 (*).

^e^ Likelihood ratio test for H_1_: ‘The Cobb-Douglas model is an appropriate functional form ‘. The significance level α = 0.05 (*).

^f^ The difference of Bayesian information criterion (BIC) values of the stochastic frontier Cobb-Douglas and translog production models. Positive values favor Cobb-Douglas in every case; values over ten indicate a very strong evidence against translog (*).

#### Stochastic frontier Cobb-Douglas production model

In the stochastic frontier production models, where a new term, the technical inefficiency of farms was included, the null hypothesis, H_0_: *γ* = 0 (the technical inefficiency effect in the model is zero), was rejected (P<0.002) for all the years (apart from 2000) (Table 3). Therefore, we concluded that there was a technical inefficiency effect in the model for all the years (apart from 2000), i.e., the farms differed from each other in terms of resource-use efficiency, and therefore the stochastic frontier models fitted to the data better than the regression models. The exponential distribution for the technical inefficiency term was found to be more appropriate than the half-normal and truncated normal distributions, based on the information criteria. However, the differences among the selected distribution and the other ones considered were minor.

According to the BIC criterion, the Cobb-Douglas stochastic frontier model was adequate for every year. Similarly to the regression model, the fittest stochastic frontier production model showed no statistical support for the negative dependence of resource-use efficiency on diversity: p-values of the Shannon index varied from 0.58 to 0.99 over years (Table 4). The mean technical efficiency score of farms, 0.8, indicated that the average resource-use efficiency of the farms was 80%.

**Table 4:** Maximum-likelihood estimates of the land-use diversity of the stochastic frontier Cobb-Douglas production models.

Year	Coefficient of Shannon index	Standard Error of Shannon index	P value of Shannon index
2000	0.018	0.113	0.873
2001	0.018	0.101	0.860
2002	0.002	0.111	0.990
2004	-0.063	0.115	0.582
2005	0.055	0.102	0.592
2006	-0.037	0.097	0.701

The small coefficient of the Shannon index with no statistical significance indicates no or a minor dependence of resource-use efficiency on land-use diversity. Shannon index = Shannon index for land-use diversity.

#### Stochastic frontier translog production model

Based on the alternative comparison method, likelihood ratio test, the Cobb-Douglas stochastic frontier production model was an adequate functional form for the years 2004 and 2006 (Table 3). For the years 2001, 2002 and 2005, the more flexible translog production model was more appropriate. In the years 2001, 2002 and 2005, a small positive, statistically non-significant, dependence of resource-use efficiency on land-use diversity was indicated p = 0.406, p = 0.663 and p = 0.560, respectively) (Table 5).

**Table 5:** Maximum-likelihood estimates of the stochastic frontier translog production models for years 2001, 2002 and 2005.

	2001		2002		2005	
Variables	Coefficient	SE	Coefficient	SE	Coefficient	SE
Intercept of production line						
Cereals, oilseeds and protein crops	-0.238 ***	0.054	-0.243 ***	0.065	-0.146 **	0.073
Field crops and grazing livestock	-0.014	0.071	-0.205 **	0.093	-0.005	0.098
Field crops	0.302 ***	0.063	0.185 ***	0.064	0.403 ***	0.080
Specialist granivores	0.728 ***	0.076	0.622 ***	0.090	0.905 ***	0.099
Specialis dairying	0.397 ***	0.065	0.304 ***	0.064	0.418 ***	0.076
Various crops and livestock	0.481 ***	0.065	0.420 ***	0.071	0.619 ***	0.076
**Shannon**	**0.083**	**0.099**	**0.047**	**0.109**	**0.062**	**0.107**
UUA	0.244 ***	0.068	0.190 **	0.077	0.263 ***	0.079
Labour	0.227 ***	0.057	0.261 ***	0.055	0.312 ***	0.056
Capital	0.621 ***	0.050	0.674 ***	0.053	0.601 ***	0.058
UUA*UUA	0.924 ***	0.210	0.852 ***	0.248	0.265	0.254
Labour*Labour	0.102	0.115	0.193 *	0.102	0.047	0.103
Capital*Capital	0.705 ***	0.130	0.639 ***	0.138	0.395 ***	0.147
Shannon*Shannon	1.281 **	0.583	2.741 ***	0.748	1.876 ***	0.641
UUA*Labour	0.090	0.109	0.113	0.117	-0.126	0.116
UUA*Capital	-0.816 ***	0.132	-0.656 ***	0.137	-0.377 ***	0.144
Labour*Capital	-0.158	0.106	-0.324 ***	0.117	-0.139	0.116
Shannon*UUA	-0.566 **	0.263	-1.054 ***	0.286	-0.584 **	0.287
Shannon*Labour	0.013	0.166	-0.012	0.195	0.062	0.174
Shannon*Capital	0.402 *	0.209	0.676 ***	0.216	0.399 **	0.198
σ_v_	0.206 ***	0.019	0.254 ***	0.019	0.280 ***	0.021
σ_u_	0.304 ***	0.030	0.230 ***	0.031	0.276 ***	0.034

All variables are mean-corrected to zero, which implies that the first-order estimates of the model represent the corresponding elasticities. All elasticities of Shannon are positive and statistically non-significant at the level of 5%.

*^,^ **^,^ *** Statistically significant at 0.10, 0.05 and 0.01 level, respectively.

Shannon land-use diversity; UAA = utilised agricultural area.

**Fig 2:**
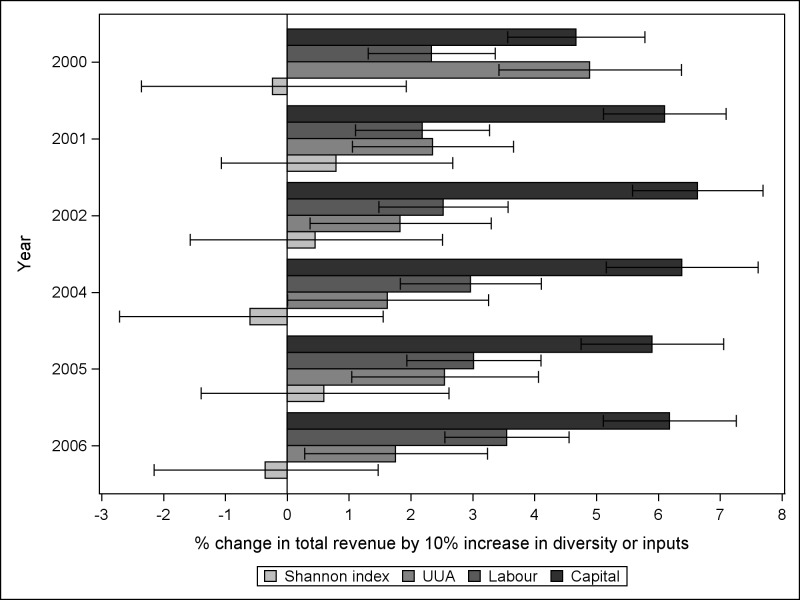
Dependence of resource-use efficiency on land-use diversity. Estimated elasticities of land-use diversity and inputs in years 2000–2002 and 2004–2006 summarised based on the results of the adequate model for each year. The elasticities illustrate the dependence of resource-use efficiency on land-use diversity and inputs. The small negative elasticities of in 2004 and 2006 indicate that a 10% increase in land-use diversity, with an average input use, would result in approximately half a percentage decrease in total revenue. The positive elasticities indicate increase in total revenue by increasing inputs. UAA = utilised agricultural area.

According to the translog production model, the coefficients of the interactions of land-use diversity with UUA and capital indicated that as land-use diversity of a farm increases, the resource-use efficiency of UUA use tends to decrease and the resource-use efficiency of capital use to increase, in terms of revenue [63]. There was no statistically significant interaction of land-use diversity and resource-use efficiency in labour use (Table 5). The elasticities of inputs of both Cobb-Douglas and translog models could be interpreted similarly, because all the variables were divided by their sample means before estimation. The elasticities illustrate the dependence of resource-use efficiency on land-use diversity and inputs. The small negative elasticities of land-use diversity in 2004 and 2006 indicated that a 10% increase in land-use diversity would result in approximately half a percentage decrease in total revenue. The positive elasticities indicated increase in total revenue by increasing inputs (Fig 2). The confidence intervals indicate that total revenue could at maximum increase by 3% (year 2001) or decline by 3% (year 2004) associated to 10% increase in land-use diversity (Fig 2).

The lack of statistical significance in the dependence of resource-use efficiency on land-use diversity, together with the near-zero value of the coefficient for the land-use diversity, indicate that there is either no dependence of resource-use efficiency on land-use diversity or the dependence is very small.

## Discussion

### Dependence of farm productivity on diversity

According to our analysis, resource-use efficiency, i.e., the revenue per unit of land, labour and capital, had no relation or only a negligible positive or negative relation with land-use diversity. Consequently, the results did not support our hypothesis of an inverse dependence between farm resource-use efficiency (critical for economic revenue as well as for eco-efficiency) and diversity (critical for resilience). The slight relations observed were inconsequent and weak enough to suggest a low economic significance for the relation in practice.

Tilman et al. [63] showed, however, based on theoretical models, that the primary productivity of ecosystems and the efficiency of resource use should be positively associated with diversity. Contrary to the general belief driving the dominant development of agricultural landscapes, the ecosystem should not only become more temporally and spatially stable [63] as diversity increases, but would also give greater yields and represent greater economic value in a resource-limited situation than is possible given lower diversity.

Tilman et al. [63], p. 416 suggest that *in a homogeneous and unchanging environment*, *the value of diversity arises because sampling from more species or industries allows a better fit between the winning species or industry and environmental conditions*. *In heterogeneous environments*, *diversity is of value because an array of options increases the goodness of fit between species traits and environmental conditions*. *Thus greater diversity allows more complete spanning of potential ecosystem or market niches*. Their results show that decreased local diversity can lead to lower production (in accordance to [17]) and lower efficiency in use of limiting resources. This relation did not hold for land-use diversity in our empirical case of the farms with an average or larger land area in southern Finland. This was the case neither within a time span of one year only which exemplifies the significance of diversity as a response to spatial but not to temporal variation, nor when a longer period of six years was considered.

Fletcher and Hilbert [64], also through a theoretical modelling exercise, showed that the opportunity cost of resilience in landscape exploitation varied greatly depending on the management strategy. They emphasized that a given stable and sustainable equilibrium point in an exploited system will be more or less resilient depending on the exploiter's strategy, because of multiple steady states in the nonlinear systems. These multiple steady states [4] give rise to the potential for a discontinuous resilience threshold, with resilience and production maintained on one side and greatly degraded on the other. Such variation in management strategies among farms may have contributed to our results of an inconsistent relation between diversity as a key determinant of resilience, and resource-use efficiency.

Our results are in accordance with the theoretical conclusions of Fletcher and Hilbert [64]: adopting, e.g., diversification as a strategy to enhance resilience does not exclude relatively high resource-use efficiency in terms of economic revenue. This insight emphasizes the potential of adaptive management in farming to simultaneously embrace both of the essential dimensions of sustainability, i.e., resilience and effectiveness [65]. Agricultural management practices benefitting multiple goals [66–67], such as resilience and resource-use efficiency, are increasingly needed to sustain food security in the face of resource scarcity and uncertainty caused by climate change and market volatility.

### Reliability and generality of the findings

The dominant discourse of sustainable development focuses on eco-efficiency, with resource-use efficiency as an important precondition. This study related another dimension of sustainability with increasing importance, resilience, to efficiency of generating revenue by use of the most important natural and economic agricultural resources: land, labour and capital. The total farm revenue is also closely related to the output of material goods, i.e., the provisioning ecosystem service of food production [68]. As the product prices and subsidy level are practically equal for all studied farms that have a similar location and size class in the years of stable price development, the revenue is also linearly related to food output among farms within a production line. Therefore, also the price level hardly interferes with the relation between diversity and resource use efficiency in terms of farm revenue within production line. Thus, this estimate of resource-use efficiency can be considered a representative indicator, representing the efficiency in use of natural and economic resources, in terms of food supply and economic revenue.

Functional diversity in landscapes contributes to supporting, regulating, provisioning and cultural ecosystem services [47,66,68]. Diversity in agricultural land-use, however, generates not only functional diversity, but may also provide response diversity associated with resilience to turbulence [20,23]. Ecological, institutional, and livelihood diversity may be among the least controversial determinants of resilience, indicating both reduced sensitivity of a system to disturbance (e.g. [69–71]) and enhanced ability to recover from and adapt to abrupt changes that are difficult to predict, i.e., adaptive capacity [72–73]. Specifically, diversity of agricultural land-use reduces vulnerability and has thus been linked to the resilience of social-ecological systems [45,51, 74–75]. Land-use diversity encompasses both the ecological and economic dimensions of farm resilience, along with socio-cultural and other dimensions [76] and is annually managed by every farm.

Farms in southern Finland represent the most northern and thus climatically vulnerable agriculture in the world. Since climate change is especially rapid in the Northern areas, Finnish farms may face the most rapid climate change of any farms in the world, with all attendant direct and indirect impacts and a high degree of uncertainty with various ecological, livelihood and knowledge-related sources [3,76–77]. Finnish farms are also tightly linked with volatile regional and global food markets. The focus on farms representing the main production lines at these northern latitudes and land area exceeding 30 ha, ensures the relevance of the case for land-use and food production. The slight increase in land-use diversity along with farm size is in accordance with the findings of McNamara and Weiss [78]. By restricting the case by region and by size class, we avoided bias from the dependence of diversity and revenue per resource unit on the latitude or size. Such simultaneous dependence could be erroneously interpreted as dependence between diversity and resource use efficiency, while being an artefact.

## Conclusions

We conclude that diversification, as a strategy to enhance resilience in agriculture, hardly constrains resource-use efficiency. This conclusion contradicts the assumption of an unavoidable trade-off between efficiency and diversity, which has driven the continuous decline in the diversity of agricultural systems. Consequently, striving for resilience is not necessarily opposed even to short-term economic efficiency.
